# Temperature and Bias Dependence of the DC and RF Characteristics of a 150 nm AlGaN/GaN-on-SiC HEMT for Microwave Applications

**DOI:** 10.3390/mi17080874

**Published:** 2026-07-23

**Authors:** Mohammad Abdul Alim, Christophe Gaquiere

**Affiliations:** 1 Electrical and Electronic Engineering, University of Chittagong, Chittagong 4331, Bangladesh; 2 Institute of Electronic, Microelectronic and Nanotechnology (IEMN), The University of Lille, 59000 Lille, France; christophe.gaquiere@iemn.univ-lille1.fr

**Keywords:** 150 nm GaN HEMT, thermal variation, multi-bias, DC parameters, RF performance, circuit parameters

## Abstract

We examine the bias- and temperature-dependent direct current, radio frequency, and equivalent-circuit characteristics of a 150 nm AlGaN/GaN/SiC high-electron-mobility transistor (HEMT) for high-frequency applications. DC and S-parameter measurements on the wafer were conducted under various gate-bias settings within a varying thermal condition (−40 °C to 150 °C). The findings suggest a distinct decrease in drain current and transconductance with rising temperature, mostly attributed to heightened carrier dispersion and self-heating effects. At V_ds_ = 15 V, the I_ds_ decreases from 247.63 mA at −40 °C to 142.94 mA at 150 °C. The maximum transconductance decreases from approximately 56.5 mS to 31.5 mS. The assessed thermal resistance varies from 6.3 °C·mm/W to 11 °C·mm/W, signifying an increasing thermal limitation at elevated temperatures. The device has a point at which the temperature coefficient is zero at V_gs_ = −7.0 V, where the threshold-voltage shift and mobility degradation counterbalance one another. Small signal investigation indicates that f_t_ decreases from approximately 53 GHz to 39 GHz, whereas f_max_ declines from 107 GHz to 74 GHz within this temperature range. The derived equivalent-circuit characteristics demonstrate the temperature sensitivity of intrinsic capacitances, resistances, transconductance, and delay components, but extrinsic capacitances and inductances exhibit comparatively lower temperature sensitivity. The measured and modeled S-parameters are in strong agreement, hence validating the extraction methodology. The findings may serve as valuable guidance for bias optimization and thermally conscious RF circuit design with GaN HEMT technology.

## 1. Introduction

The use of gallium nitride (GaN) high electron mobility transistors (HEMTs) on silicon carbide (SiC) substrates is widespread in high-frequency and high-power electronic systems. Some examples of these systems are microwave communication, radar, and power amplification. GaN/SiC HEMTs can provide lower conduction loss, higher breakdown voltage and better heat dissipation than conventional Si, GaAs and other lower-bandgap technologies, which are essential for compact RF and microwave systems. AlGaN/GaN and related heterostructures on Si and SiC, enable superior power and RF performance versus Si and GaAs, but require careful substrate choice, buffer design, trap/passivation engineering, field-plate structures, and accurate modeling to manage reliability and parasitics [1,2,3,4,5,6,7,8,9,10,11,12].

However, the performance of GaN HEMT is very sensitive to temperature and bias conditions. Self-heating can raise the channel temperature during operation, causing mobility degradation, threshold-voltage shift, decreased drain-source current (I_ds_), and changes in small-signal RF parameters. Previous studies have reported temperature-dependent behavior in several FET and HEMT technologies [13,14,15,16,17,18,19,20,21,22,23,24,25,26,27,28,29,30,31,32,33,34,35], including DC characteristics, equivalent-circuit modeling, optimization-based parameter extraction, and sensitivity analysis [13,14,15,16,17,18,19,20,21,22,24,27,28,29,30,31,32,33,34,35]. Across GaAs, AlGaAs/GaAs, and AlGaAs/InGaAs HEMTs and pHEMTs, low and cryogenic temperatures significantly modify currents, transconductance, threshold voltage, RF gain, and noise. These changes can be captured with linear-temperature small-signal circuits or neural-network-based models, enabling accurate prediction of circuit behavior and revealing additional low-temperature phenomena such as the kink effect and stability issues in large devices [13,14,15,16,17,18,19,20,21,22]. The GaN HEMT’s RF performance consistently deteriorates at increased temperatures and the intrinsic and parasitic elements are changed, so temperature-aware models are necessary. Equivalent-circuit extraction provides physical insight and parameter trends, while neural and hybrid models provide compact, high-accuracy representations of the temperature-dependent S- and noise parameters over wide ranges [27,28,29,30,31,32,33,34,35]. However, many studies have considered DC, RF, extrinsic, or intrinsic behavior separately. Therefore, a unified analysis is still needed to connect multi-bias operation, wide temperature variation, equivalent-circuit parameters, RF figures of merit, and stability behavior within the same measurement framework.

This work aims to investigate the multi-bias and temperature-dependent performance of a 150 nm GaN HEMT from −40 °C to 150 °C. The specific objectives are to examine the variation in I_ds_, threshold voltage, transconductance, and thermal resistance under different temperature and bias conditions; extract extrinsic and intrinsic equivalent-circuit parameters from measured S-parameters; evaluate intrinsic time constants and non-quasi-static behavior; analyze RF parameters including f_t_, f_max_, S_21_, Y_21_, and stability factor K; and validate the measured results with modeled S-parameter data.

On-wafer DC and RF measurements were performed within a varying temperature condition of −40 °C to 150 °C under various gate-bias circumstances. The results demonstrate that when temperature rises, carrier dispersion and self-heating effects intensify, culminating in a decrease in drain current and transconductance. At V_ds_ = 15 V, I_ds_ drops from 247.63 mA at −40 °C to 142.94 mA at 150 °C. The peak conductance decreases from about 56.5 mS at −40 °C to 31.5 mS at 150 °C. The extracted thermal resistance has increased from 6.3 °C·mm/W to 11 °C·mm/W. This means that the thermal constraint at the higher temperature is stronger. The research finds a zero-temperature-coefficient point at V_gs_ = −7.0 V where mobility degradation and threshold-voltage shift cancel each other out. RF research shows a decrease in ft from approximately 53 GHz to 39 GHz and a decrease in fmax from approximately 107 GHz to 74 GHz in the same temperature range. The S-parameters from measurement and modeling are in good agreement and thus validate the extraction methodology.

The proposed characterization methodology should also be viewed within the broader context of GaN HEMT experimental characterization. Pulsed I–V and current-transient techniques have been widely employed to investigate trapping phenomena, gate lag, drain lag, current collapse, and dynamic ON-resistance while minimizing self-heating. These methods provide valuable insight into trap dynamics and device reliability. In contrast, the present study employs steady-state temperature- and bias-dependent DC and S-parameter measurements from −40 °C to 150 °C, enabling simultaneous extraction of DC characteristics, RF figures of merit, and intrinsic/extrinsic equivalent-circuit parameters under practical operating conditions. Thus, the proposed methodology complements transient characterization by providing experimentally validated temperature-dependent parameters suitable for compact modeling, RF circuit design, and thermal-aware MMIC simulation. Recent studies by Angelotti et al. [36] on trapping dynamics in 150 nm/100 nm GaN HEMTs and Painter et al. [37] on thermal resistance further highlight the importance of combining transient and steady-state characterization for comprehensive device assessment.

## 2. Fabrication and Measurements

**Figure 1:**
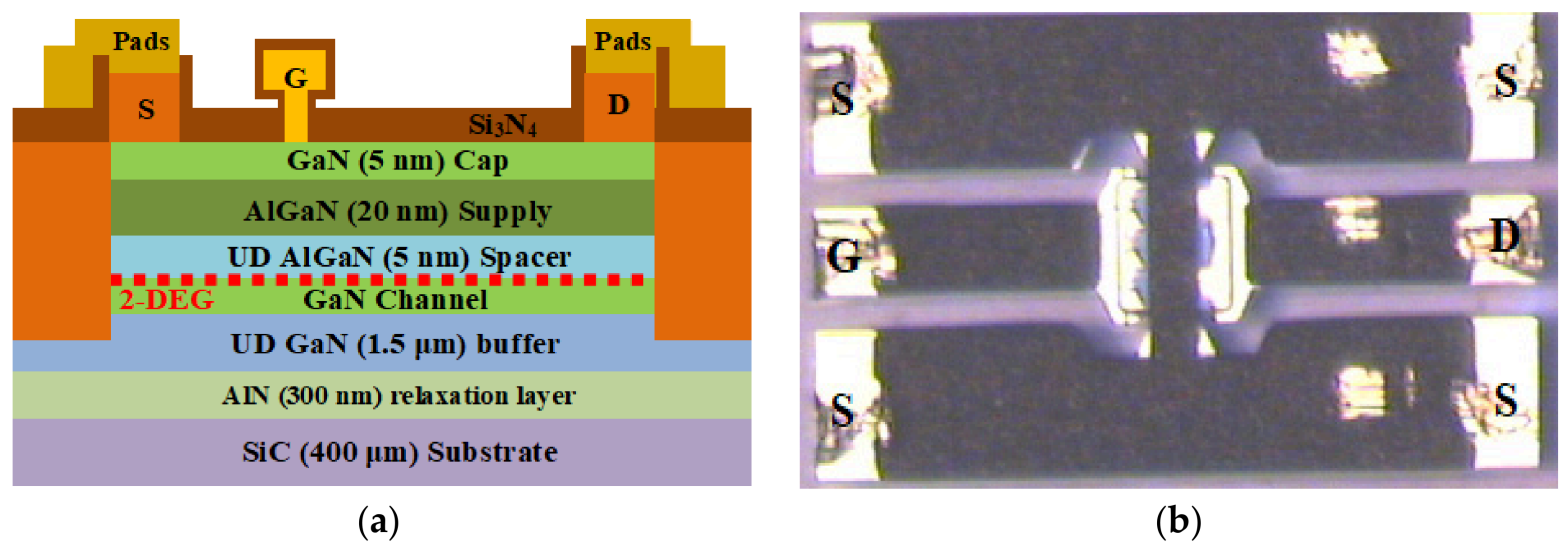
(**a**) Cross-sectional representation of the HEMT structure (not to scale); (**b**) optical mcro−graphs.

The Al_0.253_Ga_0.747_N/GaN heterostructure was produced by employing a technique known as metal–organic chemical vapor deposition (MOCVD) on a substrate composed of silicon carbide (SiC) as illustrated in Figure 1. The 400 μm SiC substrate provides mechanical support and assists heat dissipation during device operation. In order to reduce strain and get the substrate ready for the subsequent epitaxial layers, a relaxation layer of AlN/AlGaN with a thickness of 300 nm was initially produced at first. A 1.5 μm undoped GaN buffer layer was then deposited to reduce electrically active defects and improve carrier transport. The active region comprises an undoped GaN channel, a 25 nm undoped AlGaN barrier layer facilitating the development of a two-dimensional electron gas (2DEG), a 20 nm supply layer, a 5 nm spacer layer, and a 5 nm n+-GaN cap layer. Ohmic source and drain connections were fabricated using the successive evaporation of Ti/Al/Ni/Au layers with thicknesses of 12/200/40/100 nm, thereafter subjected to annealing at 900 °C. The resultant ohmic contact resistance was R_t_ = 0.36 Ω·mm. The lateral source-gate and drain-gate spacings were L_sg_ = 1 μm and L_dg_ = 2.85 μm, respectively. A 0.15 µm Schottky mushroom gate was produced by evaporation and lift-off techniques. The device features four gate fingers, each measuring 50 μm, culminating in a 200 μm overall gate width. A 240 nm Si_3_N_4_ passivation layer was ultimately created to safeguard the active surface and mitigate surface-trap effects.

**Figure 2:**
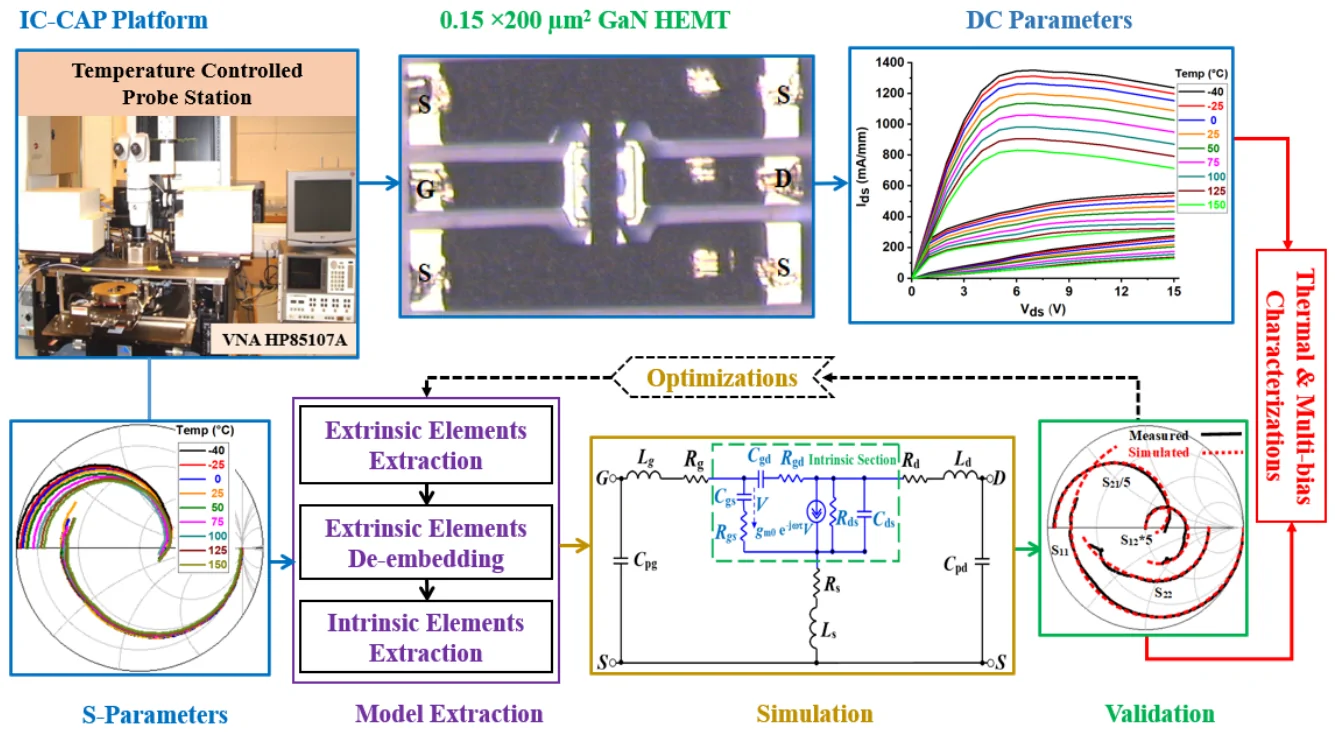
Measurement and parameter-extraction methodology used for the GaN HEMT.

Figure 2 illustrates the approach for measurement and parameter extraction utilized in the DC and RF investigation. On-wafer DC and small-signal measurements were conducted from −40 °C to 150 °C, utilizing an HP8510C vector network analyzer (Hewlett-Packard, Santa Rosa, CA, USA) and Keysight IC-CAP 2022 software [2]. A temperature control unit (Temptronic TP03200A, Temptronic, Mansfield, MA, USA) and a thermally regulated wafer chuck was employed to steady the measuring environment. At each temperature, the sample was permitted to stabilize for a minimum of 30 min to reduce thermal lag between the chuck, probes, and device. The probes were then moved to the calibration substrate, and S-parameter calibration was completed rapidly to maintain the target probe temperature. Line-line-reflect-match (LLRM) calibration was applied to define the measurement reference plane. The S-parameters were recorded between 45 MHz and 50 GHz, and the uncertainty in the temperature control was thought to be around 2 to 3%. The measured data were subsequently used for DC, small-signal, extrinsic, and intrinsic parameter extraction.

## 3. Results and Analysis

The measured GaN HEMT characteristics demonstrate three bias-dependent working zones. As V_gs_ approaches the threshold, I_ds_ increases rapidly at low gate-source voltage. As the channel opens, the current increases at intermediate V_gs_. Carrier velocity and self-heating limit current increase at higher V_gs_, causing saturation. These regions are sensitive to temperature, therefore, bias selection and thermal management are crucial for GaN HEMT device reliability.

### 3.1. DC Behavior Under Biasing Conditions and Varying Temperature

**Figure 3:**
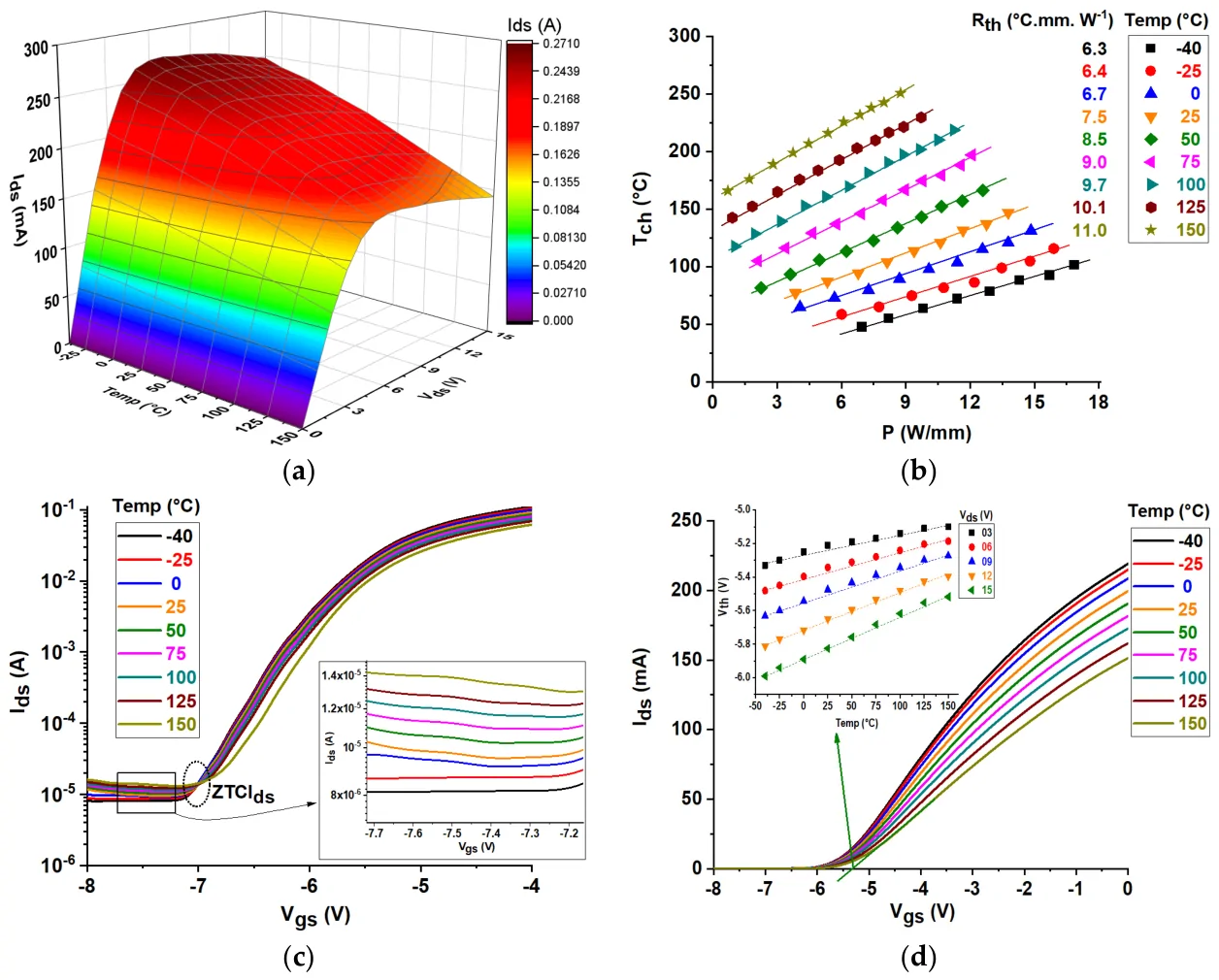
Multi-bias and thermal performance of the GaN HEMT: (**a**) DC output characteristics, (**b**) extracted thermal resistance, (**c**) transfer characteristics in semi-logarithmical representation and (**d**) threshold-voltage variation.

Figure 3a illustrates the DC output characteristics of the 150 nm GaN HEMT at V_gs_ = 0 V. For a constant V_ds_, I_ds_ typically diminishes with rising temperature due to increased carrier dispersion and reduced carrier mobility. For example, with V_ds_ = 1 V, the Ids diminishes from 75.79 mA at −40 °C to 47.58 mA at 150 °C. The decrease in current generated by temperature is exacerbated by a rise in drain bias. At V_ds_ = 15 V, I_ds_ decreases from 247.63 mA at −40 °C to 142.94 mA at 150 °C, demonstrating a significant self-heating effect during high-power operation. Although Rth derived with this method is commonly reported in the literature [38,39,40], e.g., the results are expressed in Figure 3b. Rth again increases from 6.3 °C·mm/W at −40 °C to 11 °C·mm/W at 150 °C, indicating that the thermal constraint is more significant at environment temperature. The transfer characteristics at V_ds_ = 15 V are plotted as shown in Figure 3c. From a gate voltage range of approximately −7.0 V to −8.0 V, Ids rises with temperature while Ids falls with temperature for larger zero-temperature coefficient area values of V_gs_. The semilogarithmic transfer characteristics explain this behavior. This is at the zero-temperature-coefficient point of V_gs_ = −7.0 V [41] where the threshold-voltage shift and mobility degradation nearly cancel one another out. As we can see in Figure 3d, Vth shifts to a smaller negative value with increasing temperature. The threshold voltage Vth at V_ds_ = 15 V shifts by −5.99 V at −40 °C to −5.52 V at 150 °C, which corresponds to a total shift of ~0.47 V, and hence is very important for bias stability and the design of RF circuits [42].

As shown in Figure 3d, the threshold voltage shifts from approximately −5.99 V at −40 °C to −5.52 V at 150 °C, corresponding to an average positive temperature coefficient of approximately 2.5 mV/°C. The zero-temperature-coefficient point observed near V_gs_ = −7.0 V should be distinguished from the threshold-voltage shift itself. The Vth shift changes the electrostatic turn-on condition of the channel, whereas the simultaneous reduction in carrier mobility decreases the drain current. The competing influence of these two mechanisms produces the intersection of the temperature-dependent transfer curves. Microscopically, Vth in an AlGaN/GaN HEMT is governed by the gate Schottky barrier height, conduction-band offset, polarization-induced interface charge, AlGaN barrier properties, interface or trap charge, and confinement of the two-dimensional electron gas. With increasing temperature, the different band-gap shrinkage rates of AlGaN and GaN may modify the conduction-band offset and make the quantum well at the AlGaN/GaN interface shallower. The resulting weakening of 2DEG confinement and possible change in sheet carrier concentration can contribute to the observed positive shift in Vth. Temperature-dependent changes in Schottky barrier height, interface-state occupancy, and polarization-related electrostatics may provide additional contributions. Angle-resolved X-ray photoelectron spectroscopy studies of AlNO/AlGaN/GaN structures [43] have demonstrated that interfacial band bending and potential variation must be considered when determining band offsets. Although the AlNO/AlGaN interface differs from the AlGaN/GaN heterointerface investigated here, this result emphasizes the general importance of interface electrostatics and band alignment in controlling the threshold voltage. Direct separation of these contributions would require temperature-dependent Hall or capacitance-voltage measurements, band-alignment characterization, or self-consistent Poisson–Schrödinger analysis and is therefore proposed as a subject for future investigation.

**Figure 4:**
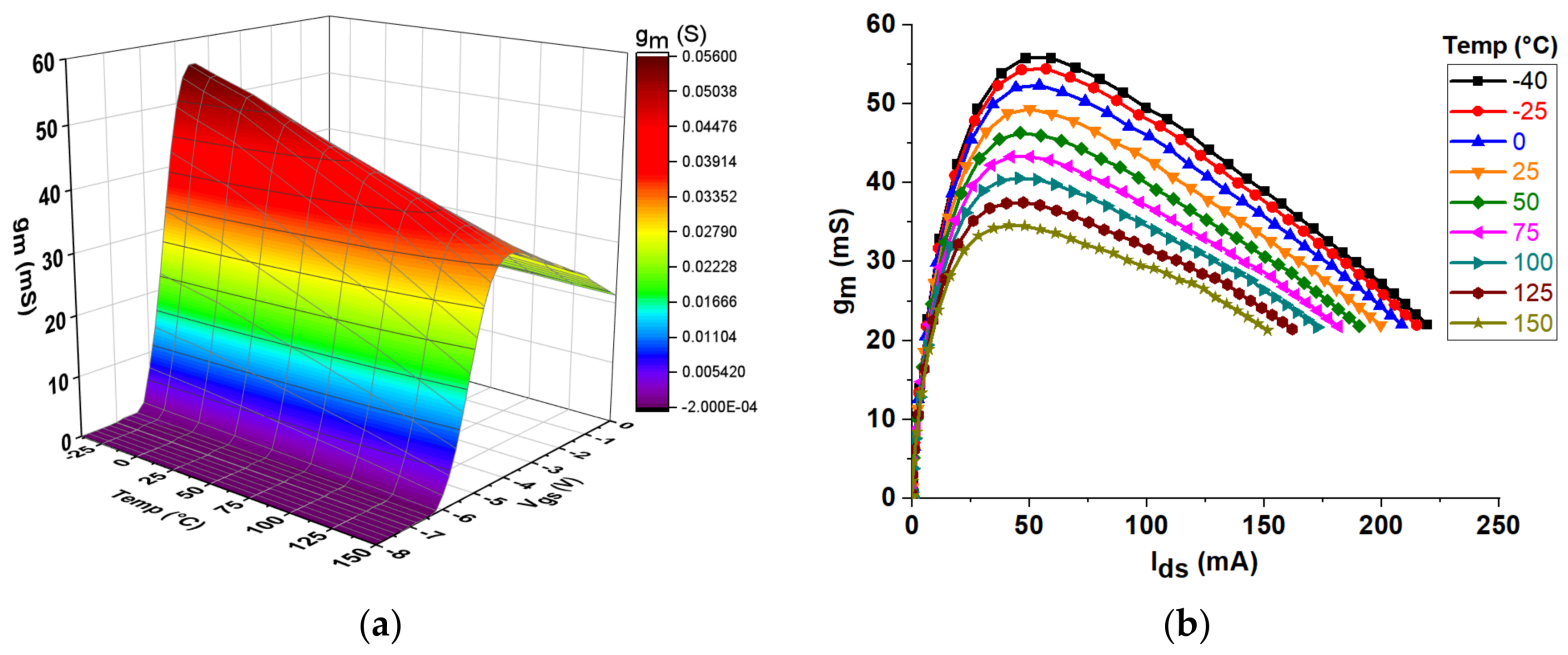
GaN HEMT multi-bias and thermal behavior: (**a**) transconductance g_m_ and (**b**) I_ds_–g_m_ relation.

Figure 4a illustrates the transconductance (g_m_) of GaN HEMT at V_ds_ = 15 V across various temperatures and gate biases. At low V_gs_ values, the device exhibits weak conduction and a minimal g_m_. As V_gs_ increases, g_m_ rises due to enhanced channel conductivity and more gate control over the drain current. The peak transconductance is approximately 56.5 mS at V_gs_ = −4.8 V and −40 °C. The maximum value diminishes to around 31.5 mS at 150 °C. The decrease in g_m_ with temperature is mostly associated with mobility deterioration and self-heating. Figure 4b similarly illustrates the temperature dependency of g_m_ as a function of I_ds_. At −40 °C, the g_m_ is around 56.5 mS with a drain-source current (I_ds_) of around 59 mA, but at 150 °C, g_m_ is approximately 31.5 mS with I_ds_ of 29 mA. The results indicate that the device has enhanced amplification capability at lower temperatures, but elevated temperatures reduce transconductance and limit RF performance.

The reduction in drain current and transconductance with increasing temperature is primarily attributed to phonon-scattering-induced carrier-mobility degradation and self-heating under high-power operation [36,37]. As the channel temperature rises, enhanced lattice scattering reduces electron mobility, thereby lowering the current-driving capability and transconductance of the device. The observed temperature dependence may also be influenced by charge trapping within the passivation/interface region and the GaN buffer. Although the investigated device employs a 240 nm Si_3_N_4_ passivation layer to suppress surface states and mitigate current collapse, interface states at the Si_3_N_4_/AlGaN interface and deep-level traps in the buffer may still undergo trapping and detrapping, particularly at elevated temperatures and under high electric-field conditions. These processes can introduce carrier dispersion and contribute to gate lag, drain lag, current collapse, and the degradation of both DC and RF performance [44]. However, because the present study is based on steady-state DC and S-parameter measurements, the individual effects of trapping, mobility degradation, and self-heating cannot be quantitatively separated. The measured temperature dependence is therefore interpreted as being predominantly governed by mobility degradation and self-heating, with trap-assisted carrier dispersion providing an additional contribution. Dedicated pulsed I–V and current-transient measurements would be required to isolate the associated trapping dynamics. Previous studies have demonstrated that optimized passivation materials, including polycrystalline AlN films, can suppress surface trapping and improve the dynamic performance of AlGaN/GaN HEMTs, emphasizing the importance of interface engineering for future high-frequency device optimization [45].

The observed thermal behavior of threshold-voltage shift and carrier mobility degradation results from the competition between two temperature-dependent physical mechanisms. Increasing temperature reduces electron mobility because of enhanced phonon scattering, which decreases the drain current and transconductance [37,46,47]. Conversely, the threshold voltage shifts toward less negative values owing to temperature-dependent changes in polarization charge, interface states, and the AlGaN/GaN heterojunction, thereby partially enhancing channel conduction. These opposing mechanisms produce the experimentally observed zero-temperature-coefficient (ZTC) bias point near V_gs_ ≈ −7 V, where mobility degradation is nearly compensated by the threshold-voltage shift. This balance is particularly important in short-channel (150 nm) GaN HEMTs, where stronger electric fields beneath the gate promote velocity saturation, self-heating, and enhanced electrothermal coupling compared with longer-channel devices. Furthermore, under the selected V_ds_ = 15 V bias, the increased lateral electric field raises channel temperature and power dissipation, making mobility degradation more dominant in the optimum transconductance region (approximately −5 V ≤ V_gs_ ≤ −4 V). Near the threshold region, however, the threshold-voltage shift partially offsets the mobility reduction, giving rise to the measured ZTC behavior. These results demonstrate that both thermal and electric-field effects must be considered simultaneously when optimizing the bias conditions of short-channel GaN HEMTs for microwave and millimeter-wave applications.

### 3.2. Extrinsic Equivalent Circuit Parameters Under Biasing and Changing Temperature

**Figure 5:**
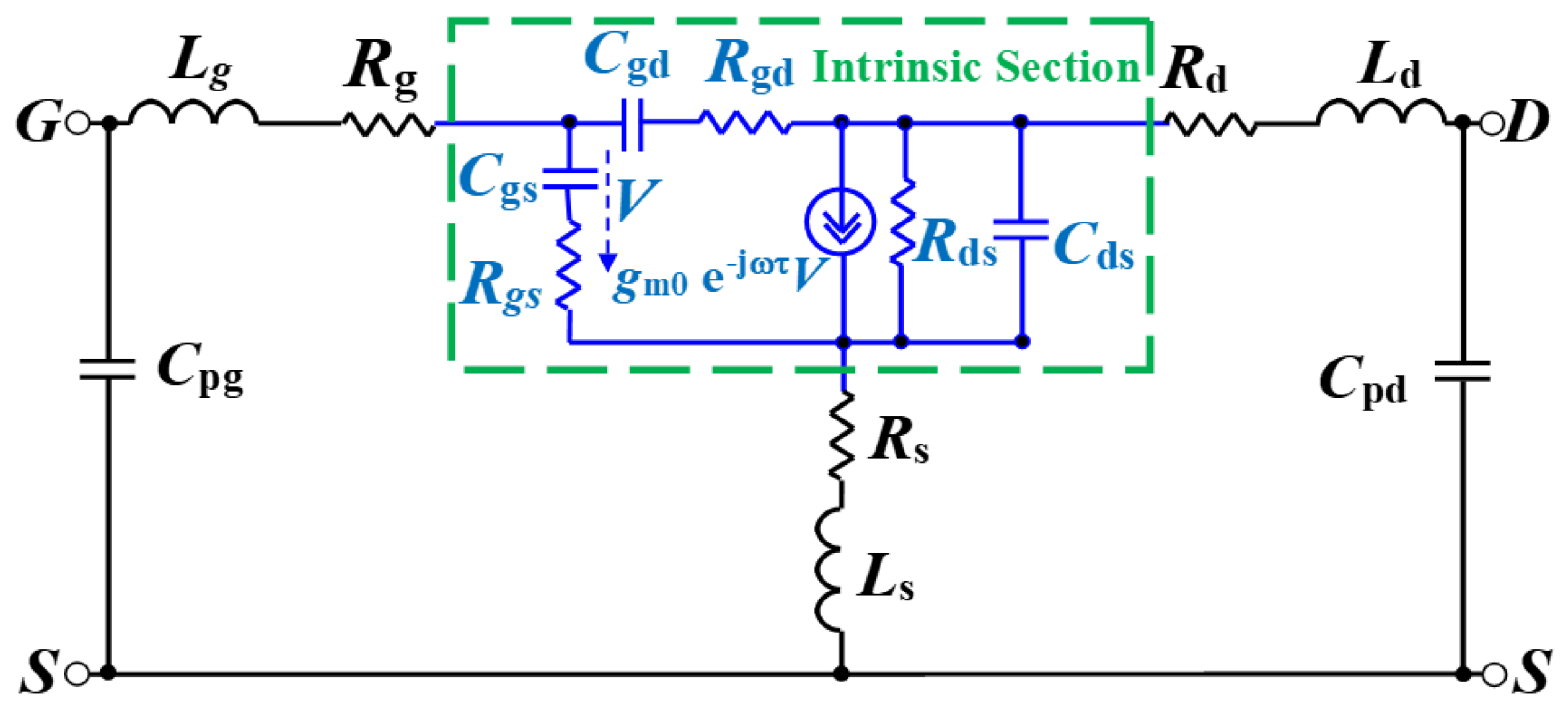
The equivalent circuit model for the investigated GaN HEMT.

**Figure 6:**
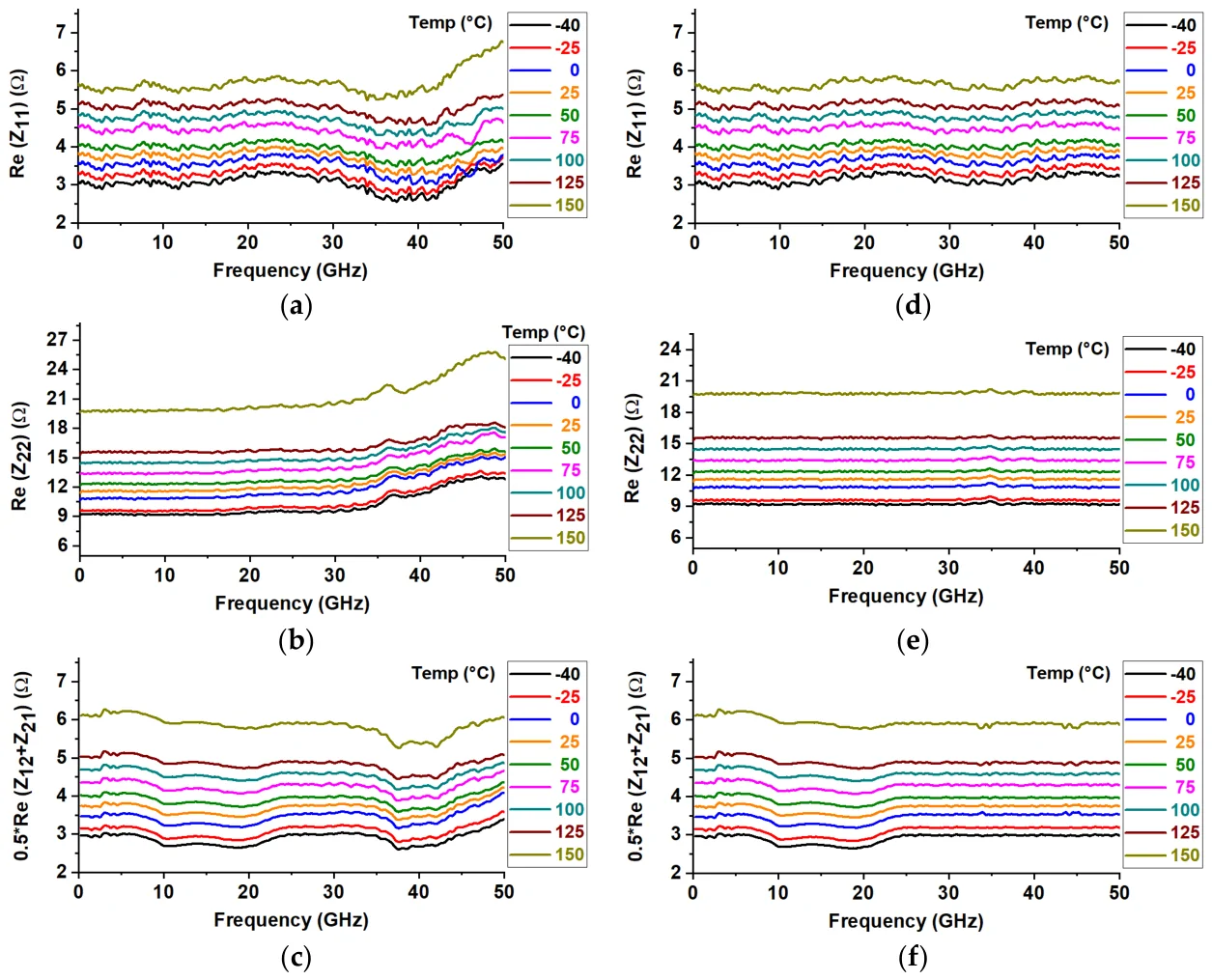
Re(Zij) of the GaN HEMT at zero bias and different temperatures: (**a**–**c**) before de-embedding and (**d**–**f**) subsequent to the de-embedding of the extrinsic capacitances.

**Figure 7:**
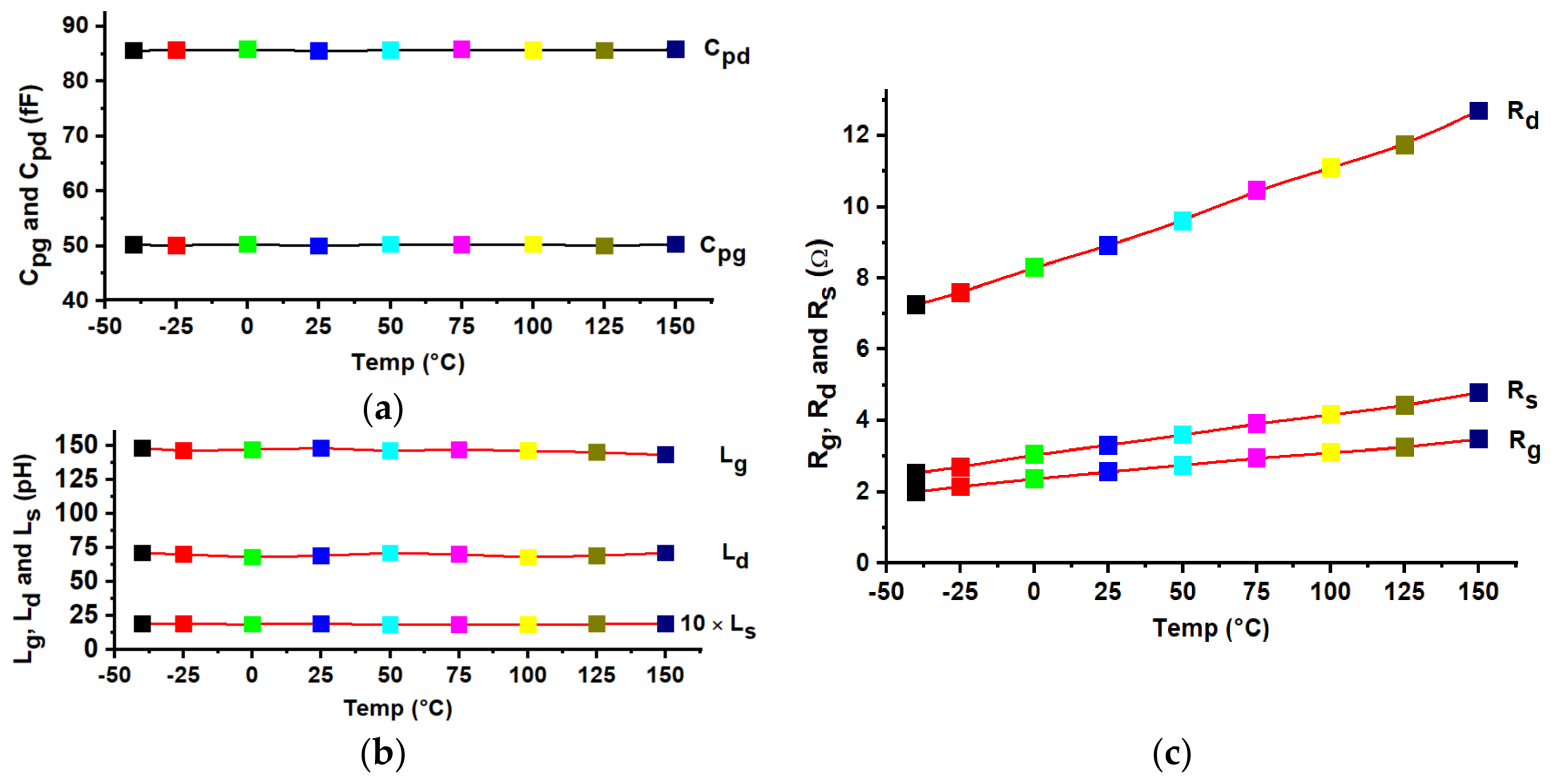
GaN HEMT’s temperature-dependent extrinsic parameters: (**a**) capacitances, (**b**) inductances, and (**c**) resistances.

Figure 5 illustrates the equivalent-circuit model of the examined device, which was analyzed to derive the S-parameters. The comparable circuit parameters were derived using the cold pinch-off approach, often employed for GaN-based HEMT technology [48]. The derived extrinsic parameters include the pad capacitances C_pg_ and C_pd_, the parasitic inductances L_g_, L_d_, and L_s_, as well as the terminal resistances R_s_, R_g_, and R_d_. Under pinch-off bias circumstances (V_ds_ = 0 V and V_gs_ = −10 V), the parasitic pad capacitances predominantly manifest in the imaginary component of the Y-parameters. Figure 6 shows the variation in Re(Zij) with temperature at zero bias and confirms the presence of the PDRZ effect [49]. The rise in Re(Zij) at frequencies above approximately 38 GHz is reduced by subtracting suitable C_pg_ and C_pd_ values during de-embedding. Figure 7a illustrates that the extracted C_pg_ and C_pd_ exhibit minimal variation with temperature, signifying a weak temperature dependence of the extrinsic pad capacitances. Under the OFF-state bias condition, the extrinsic inductances were derived from the imaginary component of the Z-parameters, and Figure 7b illustrates that L_g_, L_d_, and L_s_ exhibit minimal sensitivity to temperature. In contrast, the extrinsic resistances in Figure 7c increase almost linearly with temperature because of the access and contact regions’ temperature-dependent conductivity. The increase is most pronounced for R_d_, aligning with the extended gate-to-drain separation relative to the gate-to-source gap.

### 3.3. Equivalent Circuit Parameters Under Biasing and Varying Temperature: Intrinsic Parameters

**Figure 8:**
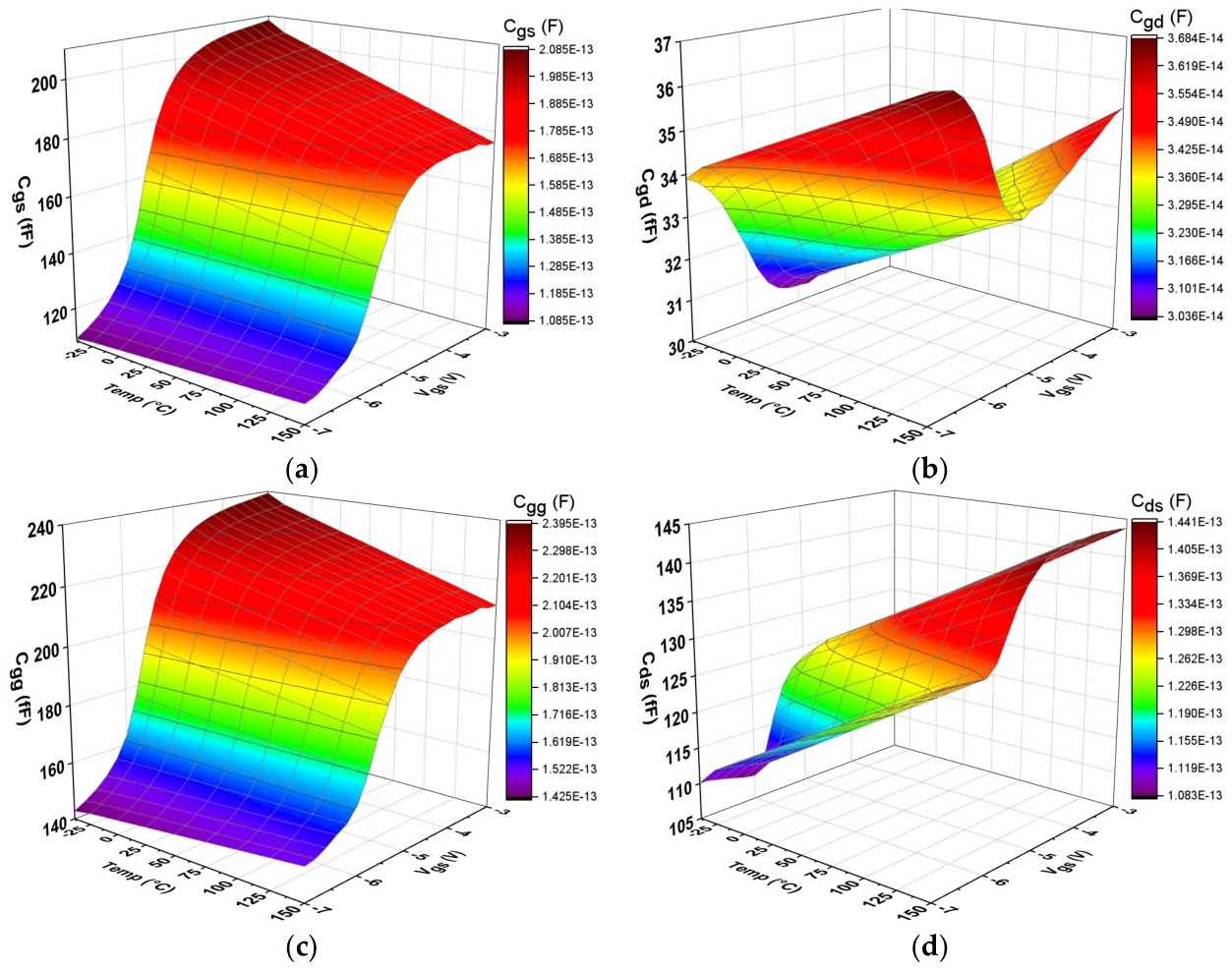
Multi-bias and thermal behavior of the GaN HEMT: (**a**) C_gs_, (**b**) C_gd_, (**c**) C_gg_, and (**d**) C_ds_.

Upon extracting the extrinsic parameters, their influence was eliminated from the measured S-parameters to derive the intrinsic device parameters (IDP). The IDP extraction was conducted at V_ds_ = 15 V, with V_gs_ values ranging from −7.0 V to −3.0 V, and across a frequency spectrum of 45 MHz to 50 GHz. This procedure separates the contributions of pad, access, and contact parasitics from the intrinsic channel behavior. A representation of the gate-source capacitance C_gs_ with temperature and voltage V_gs_ is displayed in Figure 8a. C_gs_ generally increases with V_gs_ because stronger gate bias increases channel charge control. However, at a fixed V_gs_, C_gs_ shows a moderate reduction with increasing temperature, which may be related to temperature-dependent charge distribution, 2DEG confinement, and extraction sensitivity. For example, the C_gs_ decreases from about 193 fF to 170 fF in the investigated temperature range at V_gs_ = −5 V. Figure 8b shows that C_gd_ increases with V_gs_ and it tends to increase at higher temperature. At V_gs_ = −5 V, C_gd_ increases from ~31 fF at −40 °C to 37 fF at 150 °C. Figure 8c illustrates the overall gate capacitance C_gg_, whereas Figure 8d depicts the drain-source capacitance C_ds_. The combined variation in C_gs_, C_gd_, C_gg_, and C_ds_ indicates that both bias-induced charge redistribution and temperature-dependent carrier dynamics influence the intrinsic capacitance behavior [18,50].

**Figure 9:**
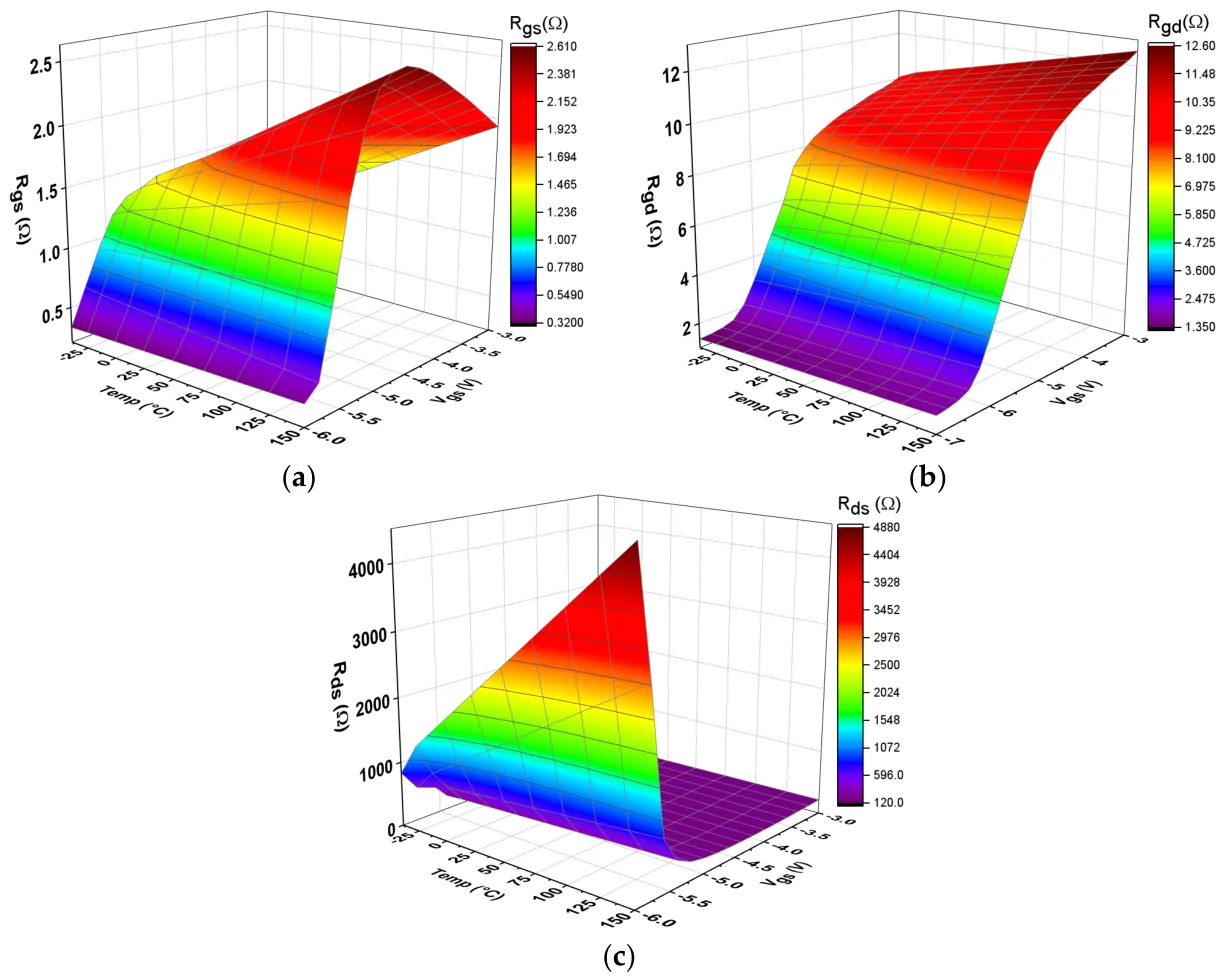
Multi-bias and thermal behavior of the GaN HEMT: (**a**) R_gs_, (**b**) R_gd_, and (**c**) R_ds_.

The C_ds_ depicted in Figure 8d represents the geometric and depletion capacitance between the drain and source terminals. At high V_gs_, C_ds_ increases because the channel is filled with more charge carriers and the depletion region is also affected. At V_gs_ = −5 V, C_ds_ increases from about 123 fF at −40 °C to 141 fF at 150 °C, and at V_gs_ = −3 V it increases from about 124 fF to 143 fF over the same temperature range. The distributed channel resistances R_gs_ and R_gd_ in Figure 9a,b are related to the real parts of the intrinsic admittance parameters and imply charging delay due to non-quasi-static effects. As V_gs_ increases from −6 V to −3 V, R_gs_ in general decreases as the channel becomes more conductive under a higher gate bias. The extracted resistance trends are dominated by a combination of bias-dependent channel charge and temperature-dependent scattering and therefore should not be attributed to carrier mobility alone. Figure 9c shows the drain-source resistance R_ds_, which is roughly proportional to 1/g_ds_. At lower V_gs_, e.g., −6 V, R_ds_ can be over 800 Ω, while it stabilizes at a much lower value of around 152 Ω for V_gs_ values near −3 V. The decrease in R_ds_ with increasing V_gs_ is a signal of better channel conduction, while the temperature dependence indicates the additional carrier scattering and resistive loss at high temperature.

**Figure 10:**
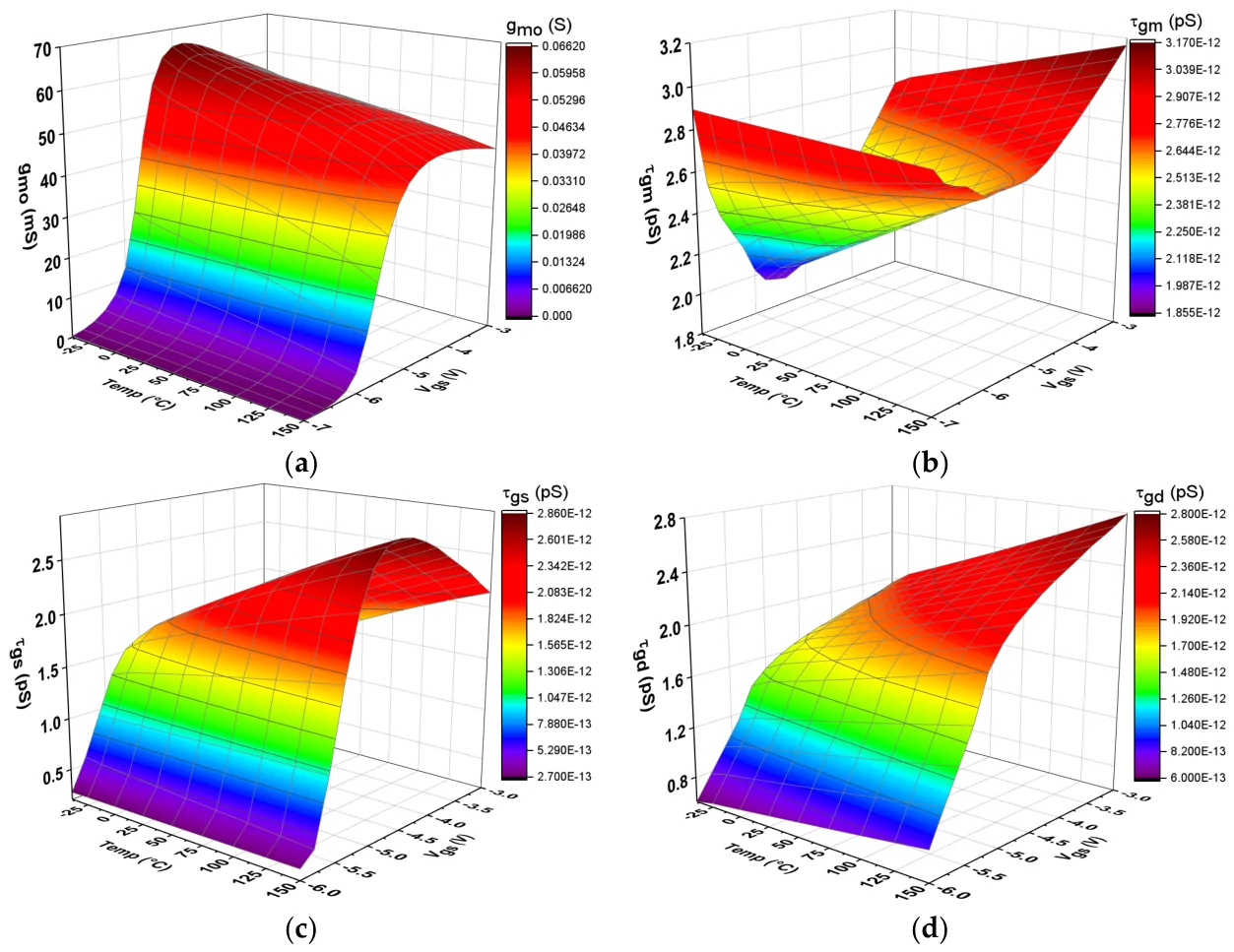
Multi-bias and thermal behavior of the GaN HEMT: (**a**) intrinsic g_mo_, (**b**) τ_gm_, (**c**) τ_gs_, and (**d**) τ_gd_.

Figure 10 shows the intrinsic transconductance g_mo_ and three intrinsic time constants (τ_gm_, τ_gs_, and τ_gd_). They are due to the finite response time of the transistor and indicate non-quasi-static effects, which gain significance at elevated frequencies [21]. For fast signal variation, with increasing V_gs_ from −6 V to −3 V, g_mo_ increases due to the enhancement of the channel conductivity and carrier density. At V_gs_ = −5 V, g_mo_ decreases from about 65 mS at −40 °C to about 43 mS at 150 °C, indicating degradation of intrinsic transconductance at high temperature. The time constants generally increase with temperature, which suggests slower charge response and therefore reduced switching speed. The delay values are relatively flat at low V_gs_. Higher gate bias and higher temperature can increase the intrinsic delay components. The findings indicate that the combined effect of bias and temperature influences the static gain capability of the device as well as the high-frequency switching dynamics.

**Figure 11:**
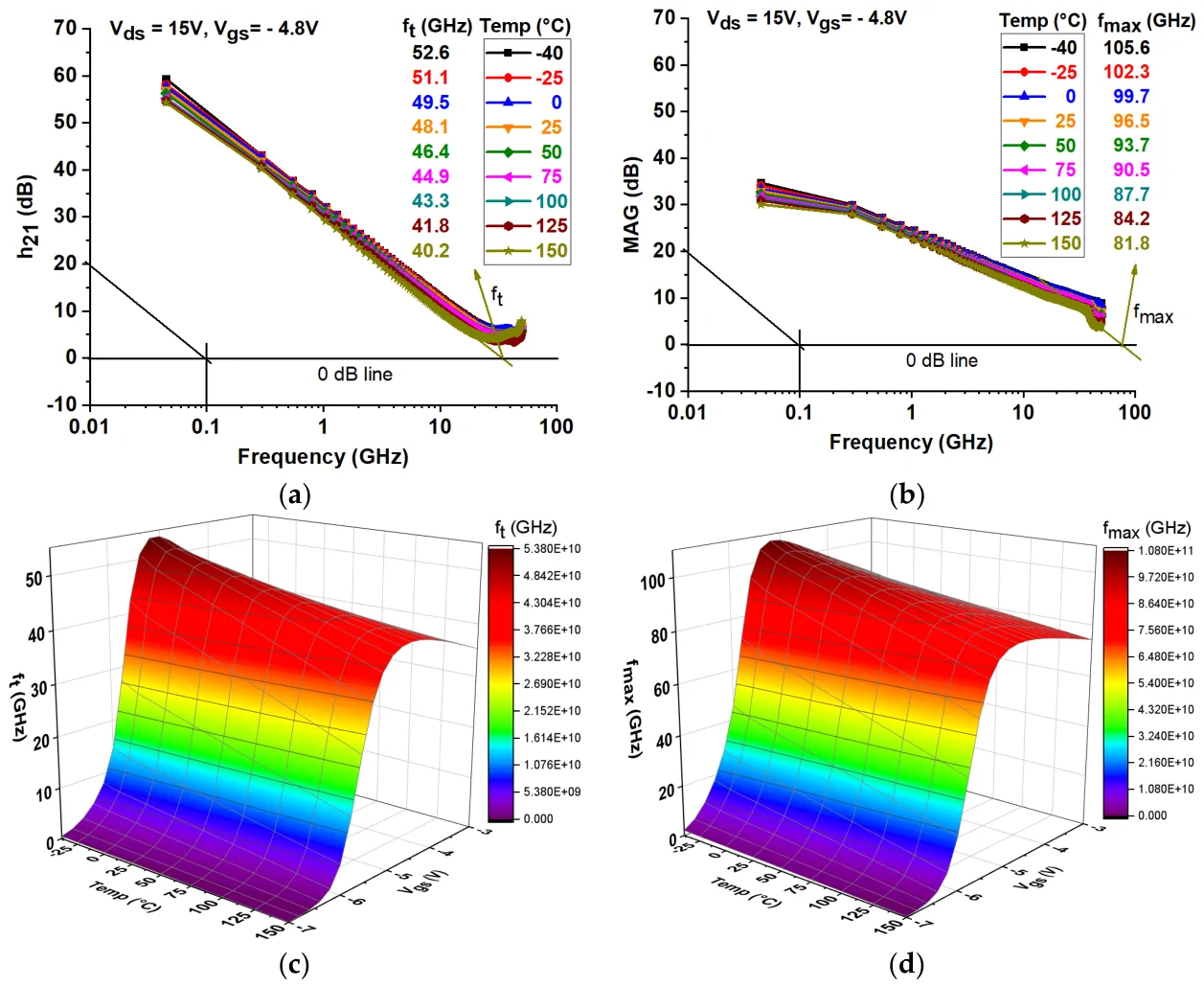
Multi-bias and thermal behavior of the GaN HEMT: (**a**) h_21_, (**b**) MAG, (**c**) f_t_, and (**d**) f_max_.

**Figure 12:**
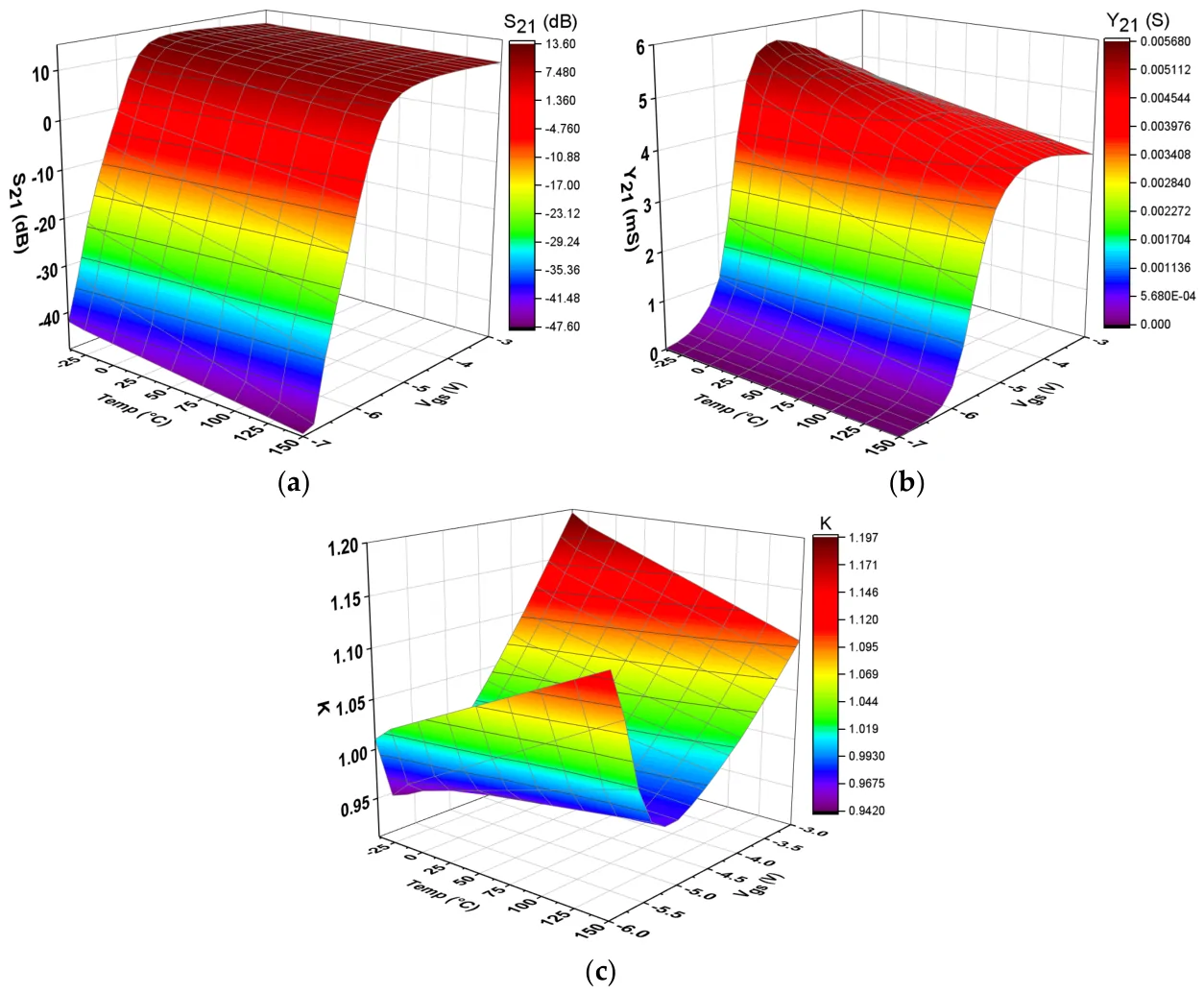
Multi-bias and characteristics of the GaN HEMT: (**a**) S_21_, (**b**) Y_21_, and (**c**) stability factor K.

In Figure 11, the measured h_21_ and MAG are shown, along with the retrieved f_t_ and f_max_. The h_21_ and MAG plots show the expected roll-off of about −20 dB/decade with frequency, although the high-frequency behavior is influenced by parasitic elements. F_t_ increases with V_gs_ sweep from −7 V to −3 V until the optimum bias region is reached. At V_gs_ = −5 V, f_t_ is approximately 53 GHz at −40 °C and decreases to approximately 39 GHz at 150 °C. Similarly, f_max_ is about 107 GHz at −40 °C and decreases to about 74 GHz at 150 °C. This reduction verifies that the RF response of the GaN HEMT is constrained by the elevated temperature through the mobility degradation, resistive loss and thermal effects. Notably, Y_21_ and S_21_ are low-frequency characteristics that have the following relationship to the intrinsic transconductance g_mo_ [51]:

(1) $$Y_{21}=\frac{g_{mo}}{1+g_{mo}R_{s}+g_{DS}\left(R_{S}+R_{D}\right)}$$

(2) $$S_{21}=-2Y_{21}\left(Z_{0}//Y_{22}^{-1}\right)$$

where Z_0_ = 50 Ω, and the approximation holds when the assumptions on the load/reference impedance render the higher-order terms of the Y-parameter negligible. In the intermediate range of bias (−5.2 V to −4.0 V), S_21_ exhibits relative stability, declining from 12.6 dB to 10.99 dB at −40 °C when V_gs_ transitions from −5.2 V to −4.0 V. At V_gs_ levels close to −3.8 V, the S_21_ displays a peak of roughly 12.77 dB at −40 °C, indicating substantial forward gearbox during low-temperature operation. Y_21_ increases with V_gs_ at the optimal transconductance area, attaining approximately 5.63 mS at −40 °C and V_gs_ = −5 V. The stability factor K is approximately equal to or greater than one across the majority of the examined bias and temperature spectra. The S_21_, Y_21_, and K (as in Figure 12) trends suggest that the device can deliver beneficial RF gain under appropriate biasing conditions; however, thermal effects diminish gain and stability margins at elevated temperatures.

**Figure 13:**
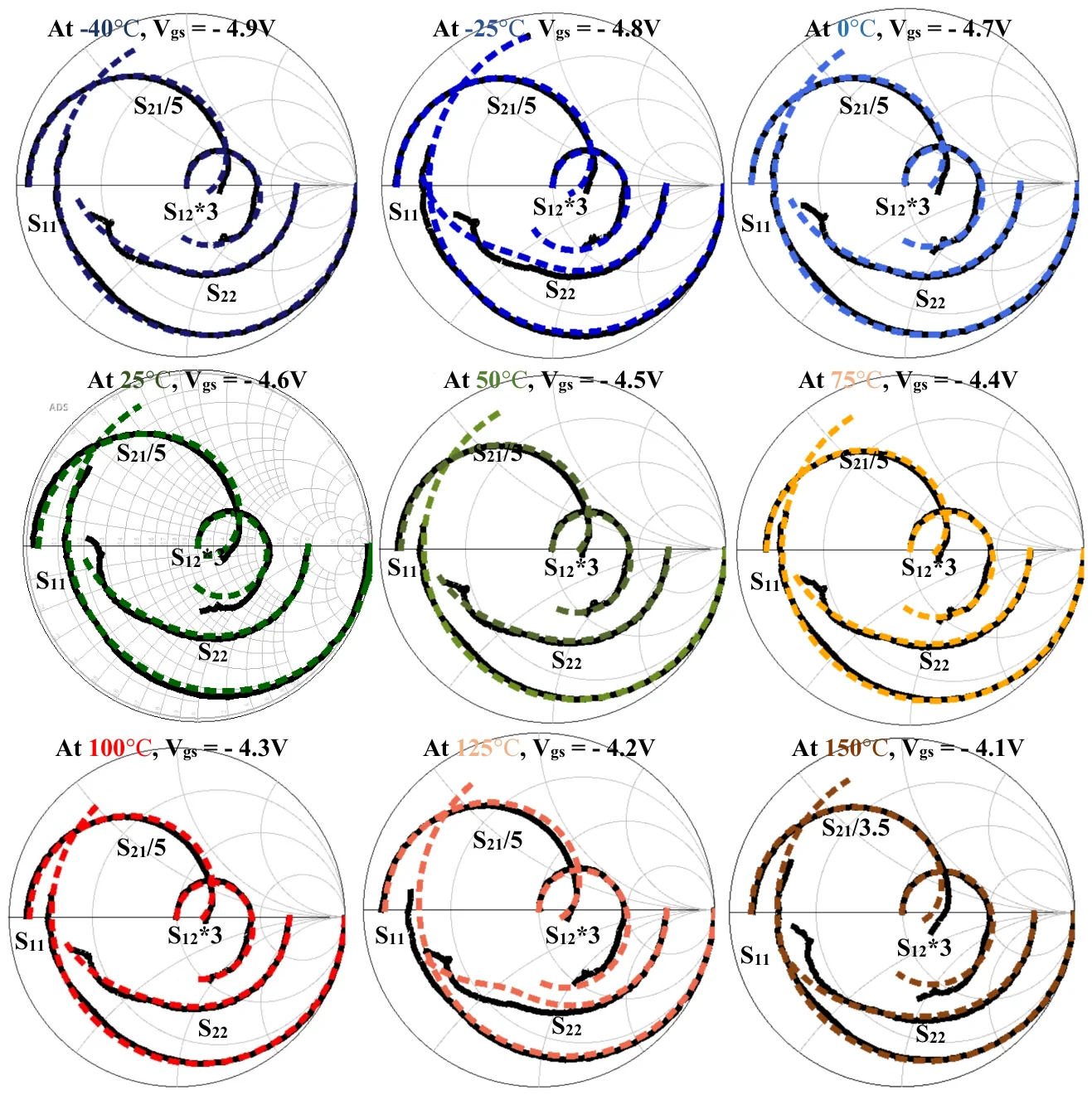
Verification of measured data (solid lines) and modeled data (dotted lines) at V_ds_ = 15 V over varying thermal conditions.

As illustrated in Figure 13, the measured S-parameters match well with the modeled data in the measured frequency and temperature ranges. This agreement attests to the reliability of the equivalent-circuit extraction routine and validates the model’s capacity to replicate the main RF behavior of the 150 nm GaN HEMT at varying thermal conditions. The direct current, radio frequency, and intrinsic parameters at V_gs_ = −4.6 V are summarized in Table 1. From −40 °C to 150 °C, I_ds_ decreases from 48.34 mA to 22.09 mA, and g_m_ decreases from 55.87 mS to 31.41 mS. The capacitances show different temperature dependencies: C_gs_ decreases and C_gd_ and C_ds_ increase moderately. The increase in the resistive components R_gs_, R_gd_ and R_ds_ with temperature is observed, which confirms the stronger resistive loss at elevated temperature. RF figures of merit also degrade with decreasing f_t_ from 51.2 to 40.6 GHz and f_max_ from 103.7 to 81.8 GHz. S_21_ decreases from 13.4 dB to 10.9 dB and Y_21_ decreases from 5.62 mS to 3.98 mS. The stability factor is close to unity but decreases slightly at higher temperature. All these trends indicate that thermal effects degrade the current drive, transconductance, gain and high-frequency capability.

## 4. Discussions and Future Directions

The findings of this study demonstrate that the DC, RF, and equivalent-circuit characteristics of the 150 nm AlGaN/GaN/SiC HEMT are significantly influenced by temperature and bias conditions. The drain current, transconductance, forward gain, and RF figures of merit are consistently diminishing from −40 °C to 150 °C. This behavior mostly pertains to the deterioration of carrier mobility, increased carrier scattering, and self-heating within the channel. Although GaN-on-SiC technology is recognized for its high power and frequency capabilities, the current findings indicate that thermal effects remain a significant constraint on stable microwave operation, particularly under elevated drain bias. The DC output characteristics show that the drain current decreases more sharply at higher drain voltages. The I_ds_ drops from 247.63 mA at −40 °C to 142.94 mA at 150 °C with a constant V_ds_ of 15 V; the very large decrease indicates that the channel is affected not only by temperature but also power-consumption mechanized self-heating effect. The thermal resistance obtained also increases with temperature from 6.3 °C·mm/W to 11 °C·mm/W over the same temperature range, thereby validating increased thermal re-striction at higher temperatures; this finding is important because high thermal resistance can constrain current-driving capability, lower gain, and accelerate reliability failure in actual RF power amplifiers. As a result, thermal management should be considered both a primary design objective and an important part of the packaging.

Transfer characterization shows a zero-temperature coefficient point centered at roughly V_gs_ = −7.0 V, where the change in threshold voltage caused by increases in temperature balances out with the degradation of mobility across this bias value. The reason for this insight is important for bias optimization, as these operations at the same current or threshold level may improve thermal stability. In practice, the device is typically biased at the peak of the transconductance region for high-gain RF operation whereupon temperature does not favor performance. Thus, there exists an authentic tradeoff between thermal stability and maximum RF gain. One of the major observations of this study is that as temperature increases, transconductance decreases. The peak transconductance reduces from about 56.5 mS at −40 °C to the value 31 mS at 150 °C; such degradation results in a reduced RF performance, as gm directly affects small-signal gain as well as moderates cut-off frequency. Also, the I_ds_–g_m_ relationship corroborates that the device can do high amplification at sub-zero temperature but has poor overall effective channel control during high-temperature operation. Such behavior agrees with the expected deterioration of mobility and increase in resistive loss due to thermal stress applied on GaN HEMTs.

Each of these extrinsic equivalent circuit characteristics is sensitive to temperature in different ways. The pad capacitances and parasitic inductances have rather weak dependence on temperature, indicating that these components are mainly geometric. On the contrary, the extrinsic resistances increase roughly linearly with temperature. This increase is primarily related to the drain resistance arising from the extended gate-to-drain access region. However, both access resistance and contact-related losses are significant at higher-temperature levels and need to be carefully addressed for correct RF model extraction and circuit simulation. Intrinsic parameters provide better understanding of the physical behavior of the device. Gate-source capacitance decreases with higher temperature, while gate-drain and drain-source capacitance increases slightly. These trends indicate that the intrinsic charge distribution and the depletion region are bias- and temperature-dependent. The report concludes that, since intrinsic transconductance decreases significantly at high temperatures, it supports the degradation in channel transit. The intrinsic delay characteristics also increase with temperature, indicating a slowing of charge response and the increased impact of non-quasi-static effects at high frequency. These results are especially relevant to microwave and millimeter-wave circuit design where small variations in capacitance, resistance and delay can greatly affect gain, phase response, and stability.

This observation using RF investigation verifies the direct effect of thermal degradation on high-frequency performance. In addition, f_t_ decreases from ∼ 53 GHz to 39 GHz and f_max_ reduces from around∼ 107 GHz to 74 GHz as the temperature increases from −40 °C to 150 °C. The reduction in ft is due to the fall in transconductance and variations in extrinsic capacitances. The reduction in fmax comes about as a result of increasing resistive losses and parasitic effects. The decrease in S_21_ and Y_21_ with increasing temperature conclusively shows that forward gain and trans-admittance fall under high temperature. Under most of the conditions investigated, K approaches or exceeds unity; however, the small decrease at higher temperatures points to a loss of stability margins. This is an important consideration in real amplifier design, since stable operation has to be maintained over a wide temperature range. The good correlation between the measured and modeled S parameters affords validation for this comparable circuit extraction method used in this study. This consistency suggests that the derived model reproduces the main RF characteristics of the device for a range of temperatures and bias points. This result is very useful in thermally aware circuit design, allowing circuit designers to anticipate device performance variations under actual working conditions. In addition, the DC, RF, thermal and equivalent-circuit analysis integrated in this work supplies a useful platform for bias selection, model building and reliable microwave circuit design using GaN HEMTs.

Future investigation has to build on this job in many respects. In order to more cleanly separate the self-heating effects from trapping effects, this work could be expanded upon by broader pulsed I–V and pulsed S-parameter data. This would allow one to then separately quantify the contributions of surface traps, buffer traps and thermal effects to current collapse and RF degradation. Whatever the case, the small-signal model derived can be coupled with electrothermal simulation to better understand channel temperature distribution and hot spots. It would enable better device setting, field-plate designs, and thermal sealing. Third, long term durability under high temperature and ho-pulse AC aging should be studied further in future works. More important are the parameters, such as threshold-voltage drift, contact deterioration, RF gain compression and breakdown behavior, that should be tracked through time so that changes in device characteristics during actual operation may be understood. Our extraction method is applicable to devices of arbitrary gate lengths, gate widths, substrates, passivation layers and field-plate configurations. Such computational analyses will pinpoint structural designs that yield the best tradeoff between RF performance, thermal stability and dependability. The extracted temperature-dependent parameters are finally used in compact models which can be subsequently employed for performing circuit level simulation. Such a delay model would allow designers of power amplifiers, low noise amplifiers and microwave front ends to predict performance over a broad range of operating temperatures. Future work may involve machine learning or hybrid modeling approaches in order to improve S-parameter and related circuit model parameter prediction under complicated bias heat conditions. The results of this study lay the groundwork for developing improved and more thermally reliable GaN HEMT devices for high-frequency microwave applications.

Although the present work focuses on temperature- and bias-dependent DC, RF, and small-signal equivalent-circuit characterization, the extracted parameters provide an important foundation for future nonlinear electrothermal large-signal modeling of GaN HEMTs. Future model development should combine the experimentally observed temperature dependence of intrinsic and extrinsic equivalent-circuit parameters with dynamic trapping phenomena, self-heating, field-dependent short-channel effects, velocity saturation, and nonlinear charge-storage mechanisms within a unified electrothermal framework. Such a model would improve prediction of device behavior under large-signal microwave and millimeter-wave operation, where strong electrothermal coupling and dynamic trapping simultaneously influence gain, efficiency, linearity, and reliability. Measurement-based large-signal modeling approaches that incorporate charge trapping under multiple bias conditions, such as the model reported by Gibiino et al. [45], have demonstrated significant progress. Nevertheless, additional developments are required to incorporate the coupled temperature-dependent and electric-field effects investigated in the present work. Therefore, the comprehensive temperature-dependent parameter extraction presented in this study can provide valuable experimental input for next-generation electrothermal large-signal compact models for GaN HEMTs.

## 5. Conclusions

This work investigated the bias- and temperature-dependent DC, RF and equivalent circuit behavior of a 150 nm AlGaN/GaN/SiC HEMT from −40 °C to 150 °C. The results show that high temperature strongly degrades the drain current, transconductance, gain and RF figures of merit. With V_ds_ = 15 V, the I_ds_ decreases from 247.63 mA at −40 °C to 142.94 mA at 150 °C and the peak g_m_ decreases from approximately 56.5 mS to 31.5 mS. The extracted thermal resistance increases from 6.3 °C·mm/W to 11 °C·mm/W, stressing the importance of thermal management for reliable high-power operation. The intrinsic equivalent-circuit parameters (g_mo_, C_gs_, C_gd_, C_ds_, R_gs_, R_gd_, R_ds_, τ_gm_, τ_gs_, and τ_gd_) indicate that the charge transport and high-frequency response are affected by the bias and temperature together. The RF analysis further shows that f_t_ and f_max_ drop when the temperature rises, while S_21_, Y_21_, and K indicate reduced gain and narrower stability margins at elevated temperature. The robust association between the measured and simulated S parameters substantiates the extraction methodology and endorses its application in thermally aware RF circuit design. The present study provides a practical approach to understanding and optimizing the 150 nm GaN HEMTs in realistic multi-bias and temperature conditions.

## Figures and Tables

**Table 1:** Summary of the DC, RF, thermal, and intrinsic equivalent-circuit parameters (at V_ds_ = 15 V and V_gs_ = −4.6 V) of the 150 nm AlGaN/GaN-on-SiC HEMT under different temperatures.

Temperature (°C)	I_ds_ (mA)	g_m_ (mS)	Rth (°C·mm/W)	C_gs_ (fF)	C_gd_ (fF)	C_ds_ (fF)	R_gs_ (Ω)	R_gd_ (Ω)	R_ds_ (Ω)	τ_gm_ (pS)	τ_gs_ (pS)	τ_gd_ (pS)	ft (GHz)	fmax (GHz)
−40	48.34	55.87	6.3	200.8	30.5	124	1.26	8.6	137.1	2.16	1.59	1.64	51.2	103.7
−25	46.7	54.35	6.4	197.5	30.9	126.3	1.43	8.8	144.8	2.23	1.77	1.72	49.8	101
0	44.24	52.08	6.7	194.2	31.4	128.7	1.59	9.1	152.4	2.31	1.95	1.79	48.5	98.2
25	40.75	48.8	7.5	190.9	31.8	131.1	1.76	9.4	160	2.39	2.12	1.87	47.2	95.5
50	37.27	45.53	8.5	187.6	32.2	133.5	1.93	9.6	167.7	2.46	2.28	1.95	45.9	92.8
75	33.78	42.26	9.0	184.3	32.7	135.9	2.1	9.9	175.3	2.54	2.43	2.03	44.6	90
100	30.29	38.99	9.7	181	33.1	138.2	2.26	10.2	182.9	2.61	2.58	2.12	43.2	87.3
125	26.19	35.19	10.1	177.7	33.5	140.6	2.43	10.5	190.6	2.69	2.72	2.2	41.9	84.6
150	22.09	31.41	11.0	174.4	34	143	2.6	10.7	198.2	2.77	2.85	2.29	40.6	81.8

## Data Availability

The data presented in this study are available on request from the authors.
